# A Novel SCN1A Mutation Associated With Reflex Seizures Induced by Movements

**DOI:** 10.7759/cureus.46702

**Published:** 2023-10-09

**Authors:** Chao Gong, Qing Li, Xuemei Li, Xiaoli Yu, Dong Li

**Affiliations:** 1 Department of Neurology, Tianjin Children’s Hospital, Tianjin, CHN

**Keywords:** gain-of-function, scn1a mutation, genetics, movement-induced reflex epilepsy, seizures

## Abstract

A 14-year-old male patient was admitted to the hospital due to epileptic seizures, which occurred at the beginning of running exercise after being stopped and fast walking. Seizures were consistently characterized by a dystonic posture of the distal portion of the left arm-flexed and adducted by the chest without loss of consciousness. We suspected that this was movement-induced reflex epilepsy and performed whole exome sequencing. Whole exome sequencing revealed a novel SCN1A missense mutation, c.5549T>G (p.Ile1850Ser). SCN1A mutations have not been reported in patients with reflex epilepsy induced by movement. This report enriches the genotypes and phenotypes of SCN1A-related epilepsy and provides further insight into the etiology of reflex epilepsy induced by movement.

## Introduction

SCN1A is a gene that encodes the alpha subunit of the voltage-gated sodium channel Nav1.1. This gene is located on chromosome 2q24.3, and mutations in SCN1A have been linked to a number of different neurological disorders, including epilepsy and autism spectrum disorder [1]. The Nav1.1 channel is primarily expressed in inhibitory interneurons in the brain, where it plays a critical role in regulating neuronal excitability. So far, more than 1200 SCN1A gene mutations have been identified [2]. Whole exome sequencing is valuable in diagnosing certain neurological disorders, such as generalized epilepsy with febrile seizures plus (GEFS+) and Dravet syndrome (DS), which are associated with mutations in SCN1A. Mutation analysis of the SCN1A gene is helpful in finding the cause and guiding the selection of antiepileptic medications.

## Case presentation

**Figure 1:**
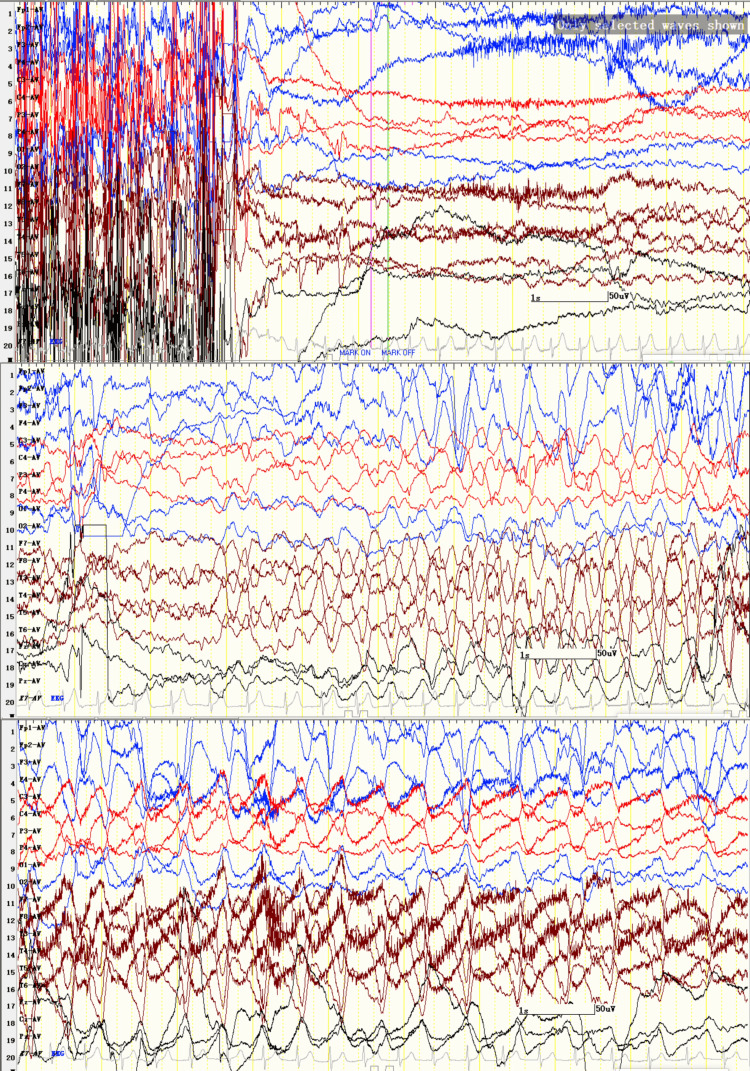
Ictal section of the video-electroencephalogram (EEG) Ictal section of the video-EEG (Sens 150uv/cm; HF 70Hz; TC 0.3s; 10sec/screen) depicted generalized low-voltage induced by the leg-raising test, followed by rhythmic slow waves and sharp and slow wave complexes on FP2, F4, F8, and T4 with progressively increasing voltage and slowing frequency

The patient, a 14-year-old male, presented with paroxysmal abnormalities more than four years before admission, all of which appeared at the beginning of running exercise after being seated, manifested by precordial discomfort, increased breathing and heart rate, followed by flexion and rigidity of the left upper limb without loss of consciousness, which lasted for more than 10 seconds. The symptoms all occurred while running and could be avoided without running, so the patient did not care and never sought medical treatment. The symptoms were more frequent approximately one month before admission to our hospital. In addition to sudden running, rising quickly from a chair or sharply accelerating the pace while walking can also induce similar seizures, approximately three to four times per day. His cognition and development were normal, and his medical history was not contributory, as there was no history of febrile convulsion. He had no severe disease or head injury in his history. The family medical history of the patient was unremarkable. Physical examination, brain CT and MRI (3T) were normal. Video-electroencephalogram (EEG) (Figure 1) captured three focal seizures when he ran in place. Ictal EEG was characterised by generalized low voltage, followed by rhythmic slow waves and sharp and slow wave complexes on the right frontal and anterior middle temporal areas with progressively increasing voltage and slowing frequency. Interictal EEG showed epileptiform discharges over the right frontal and anterior middle temporal areas, and there was an accentuation of discharges in sleep. Whole exome sequencing of the patient and his parents revealed that the patient had a novel missense mutation of the SCN1A gene (chr2:166848236:c.5549T>G p.Ile1850Ser), which was inherited from his unaffected mother. Mutation Taster (http://www.mutationtaster.org) and PolyPhen-2/HumVar model software (http://genetics.bwh.harvard.edu/pph2) predicted that the SCN1A mutation of the patient was deleterious. Based on clinical manifestations and EEG, the patient was diagnosed with epilepsy. The patient received oxcarbazepine, which was progressively increased to a dose of 0.6 g/day. After one year of follow-up, the patient did not experience a repeat of epileptic seizures.

## Discussion

We describe a case of epilepsy related to a novel SCN1A mutation presenting with reflex seizures induced by movements. It has not been reported that reflex seizures triggered by movements are associated with SCN1A mutations.

Reflex seizures are objectively and consistently demonstrated to be evoked by a specific afferent stimulus or by an activity of the patient. Those triggering stimuli include reading, writing, other language functions, startle, movements, somatosensory stimulation, proprioception, auditory stimuli, immersion in hot water, eating, and vestibular stimulation. Seizures induced by movements are very rare reflex seizures.

Movement-induced reflex epilepsy and paroxysmal exercise-induced dyskinesia (PKD) are difficult to identify. They both have the characteristics of recurrent, transient and rigid, and mostly occur at the initial start of movements and postural changes. However, PKD is a rare movement disorder and brain CT, MRI and EEG are normal. In addition, the most common causative gene of PKD is proline-rich transmembrane protein 2 (PRRT2), not SCN1A. Movement-induced reflex seizures are transient brain function abnormalities caused by abnormal discharge of neurons, and EEG mostly has abnormal discharge. Therefore, long-term video-EEG is useful for diagnosis.

SCN1A disorders result in a wide range of epilepsy phenotypes, including febrile seizure (FS), GEFS+, DS, SCN1A gene-related early-onset developmental epilepsy encephalopathy (DEEs), myoclonic dystonia epilepsy (MAE), infantile epilepsy with migratory focal seizures (EIMFS), and Panayiotopoulos syndrome [1,2]. In recent years, the spectrum of SCN1A disorders has been expanding, and focal seizure and hemiplegic migraine are both included. In addition, there were two cases of somatosensory reflex seizures in two children with epilepsy related to SCN1A mutations. These results imply that there is a certain correlation between SCN1A gene mutations and somatosensory reflex epilepsy. One child is a 10-month-old female patient with a de novo SCN1A heterozygote mutation C.1337A >C (p.Q422P). She had seizures with eye deviation and unresponsiveness induced by somatosensory stimuli of the face. Febrile and afebrile generalized and focal motor seizures soon followed [3]. The other child with the SCN1A variant (C.4012A >C p.N1338H) inherited from her healthy father manifested with refractory focal seizures at 11 years and reflexes to tactile stimuli causing frequent falls. Repeated brain MRI revealed a possible blurring over the left frontomesial lobe. At the age of 25 years, she underwent stereo-EEG recording of focal seizures followed by left frontomesial lobectomy. Following the surgical procedure, the patient continued to have seizures at a reduced frequency; when carbamazepine was added, she had long periods of seizure freedom [4]. Our patient is a single adolescent male with seizure onset in late childhood who presented with reflex seizures, and the SCN1A mutation was inherited from his healthy mother, which is similar to that case. However, his MRI was normal, which may be one of the reasons why his symptoms were less severe.

Previous studies found that some relatives of probands who were SCN1A mutation carriers never experienced seizures [5,6]. The SCN1A gene displays incomplete clinical penetrance, which explains the unaffected status of his mother. A study showed that the severity of phenotype and penetrance of families were related to the types of SCN1A mutations, and less severe phenotypes and incomplete penetrance were more common with missense mutations [5]. The patient manifested a less severe phenotype, and his mother carried the same mutation and was unaffected, which is consistent with the above study.

The mechanism of SCN1A gene-related epilepsy is closely related to the function of sodium channels. Voltage-gated sodium channels are complexes of an α subunit in association with auxiliary β subunits. There are nine functional voltage-gated sodium channel α subunits in humans, including Nav1.1-Nav1.9 [7, 8]. Nav1.1, encoded by SCN1A, is mainly expressed in GABAergic neurons, and Nav1.1 mutations mainly affect these neurons [7]. There are two types of functional properties in neurons with SCN1A mutations: gain-of-function (GOF) and loss-of-function (LOF) [9,10]. LOF variants and GOF variants can result in different disease phenotypes and guide the choice of effective antiseizure medication. The predominant LOF variants in the SCN1A gene cause DS, one of the most common monogenic epilepsies, and GEFS+. Instead, most SCN1A GOF variants cause a spectrum of disorders beyond the DS/GEFS+ dichotomy, which extends from mild focal epilepsies to hemiplegic migraine and severe DEE. Matricardi proposed that the non-DS/non-GEFS + phenotype may be associated with increased sodium channel function induced by GOF variants and that sodium channel blockers can control seizures by counteracting excessive channel function [4]. In this case, oxcarbazepine was effective, and we suspect that the sodium channel function change in this patient may be a GOF variant. We hope this can be verified in the future.

In 1964, a Lancet study reported five cases of motor-induced reflex epilepsy with abnormal EEG discharge [11]. They all presented with reflex seizures provoked by sudden movement, consisting usually of spreading tonic or athetoid spasms without loss of consciousness. According to the characteristics of familial incomplete penetrance in cases, the author proposed that there was a dominant gene with incomplete penetrance, but so far, no relevant gene has been reported in any literature. The case we report has similar clinical characteristics to the Lancet literature. Therefore, it is reasonable to assume that the dominant gene associated with motor-induced reflex epilepsy is SCN1A. This is the first time since 1964 that we have reported that reflex epilepsy induced by movement is related to SCN1A mutation, which is of great significance for us to further understand the mechanism of reflex epilepsy induced by movement and guide treatment. This report also contributes to an expanding clinical spectrum of SCN1A mutations. Finally, the possibility of screening for SCN1A mutations might be considered for those patients presenting with reflex seizures induced by movements.

## Conclusions

We describe a case of epilepsy related to a novel SCN1A mutation presenting with reflex seizures induced by movements. This is the first report since 1964 that reflex epilepsy induced by movement is related to SCN1A mutation, which is of great significance for us to further understand the mechanism of reflex epilepsy induced by movement and guide treatment. This report also contributes to an expanding clinical spectrum of SCN1A mutations.
